# Evaluation of reconstruction methods and image noise levels concerning visual assessment of simulated liver lesions in ^111^In-octreotide SPECT imaging

**DOI:** 10.1186/s40658-023-00557-4

**Published:** 2023-06-02

**Authors:** Emma Wikberg, Martijn van Essen, Tobias Rydén, Johanna Svensson, Peter Gjertsson, Peter Bernhardt

**Affiliations:** 1 Department of Medical Radiation Sciences, Institute of Clinical Sciences, Sahlgrenska Academy, University of Gothenburg, Gothenburg, Sweden; 2 Department of Molecular and Clinical Medicine, Institute of Medicine, Sahlgrenska Academy, University of Gothenburg, Gothenburg, Sweden; 3 Department of Clinical Physiology, Sahlgrenska University Hospital, 413 45 Gothenburg, Sweden; 4 Medical Physics and Medical Bioengineering, Sahlgrenska University Hospital, 413 45 Gothenburg, Sweden; 5 Department of Oncology, Institute of Clinical Sciences, Sahlgrenska Academy, University of Gothenburg, Gothenburg, Sweden

**Keywords:** Image reconstruction, Monte Carlo simulations, SPECT/CT, Neuroendocrine tumors

## Abstract

**Background:**

Early cancer detection is crucial for patients’ survival. The image quality in ^111^In-octreotide SPECT imaging could be improved by using Monte Carlo (MC)-based reconstruction. The aim of this observational study was to determine the detection rate of simulated liver lesions for MC-based ordered subset expectation maximization (OSEM) reconstruction compared to conventional attenuation-corrected OSEM reconstruction.

**Methods:**

Thirty-seven SPECT/CT examinations with ^111^In-octreotide were randomly selected. The inclusion criterion was no liver lesions at the time of examination and for the following 3 years. SPECT images of spheres representing lesions were simulated using MC. The raw data of the spheres were added to the raw data of the established healthy patients in 26 of the examinations, and the remaining 11 examinations were not modified. The images were reconstructed using conventional OSEM reconstruction with attenuation correction and post filtering (fAC OSEM) and MC-based OSEM reconstruction without and with post filtering (MC OSEM and fMC OSEM, respectively). The images were visually and blindly evaluated by a nuclear medicine specialist. The criteria evaluated were liver lesion yes or no, including coordinates if yes, with confidence level 1–3. The percentage of detected lesions and accuracy (percentage of correctly classified cases), as well as tumor-to-normal tissue concentration (TNC) ratios and signal-to-noise ratios (SNRs), were evaluated.

**Results:**

The detection rates were 30.8% for fAC OSEM, 42.3% for fMC OSEM, and 50.0% for MC OSEM. The accuracies were 45.9% for fAC OSEM, 45.9% for fMC OSEM, and 54.1% for MC OSEM. The number of false positives was higher for fMC and MC OSEM. The observer’s confidence level was higher in filtered images than in unfiltered images. TNC ratios were significantly higher, statistically, with MC OSEM and fMC OSEM than with AC OSEM, but SNRs were similar due to higher noise with MC OSEM.

**Conclusion:**

One in two lesions were found using MC OSEM versus one in three using conventional reconstruction. TNC ratios were significantly improved, statistically, using MC-based reconstruction, but the noise levels increased and consequently the confidence level of the observer decreased. For further improvements, image noise needs to be suppressed.

## Background

Cancer is a leading cause of death globally, with nearly 10 million deaths in 2020 [1]. Cancer incidence in that year was over 19 million. The global cancer burden can be reduced through early detection to increase the probability of survival, since early-stage cancer is more likely to respond to treatment [2]. Improved methods for detecting cancer earlier can also lower the cost of treatment [3]. As cancer spreads, a common site for lesions is the liver, partly due to its high blood supply from both the hepatic artery and the portal vein [4].

Nuclear medicine imaging with single-photon emission computed tomography (SPECT) or positron emission tomography (PET) cameras is an important technique in cancer diagnostics. For neuroendocrine tumors (NETs), the tumor burden can be visualized with somatostatin receptor scintigraphy, planar and SPECT/computed tomography (CT) imaging, using ^111^In-labeled octreotide. Octreotide is a somatostatin analogue that binds with high affinity to somatostatin receptors that are often highly expressed in NETs [5, 6].

The image quality achieved with nuclear medicine imaging, and with SPECT/CT in particular, is limited. The reasons are poor statistics (due to restrictions in the radiation dose to the patient and a limited acquisition time) combined with the physical properties of the photons, such as attenuation and scattering in the body, and the camera design. To try to correct for the point spread function and the collimator response, collimator detector response (CDR) modeling is nowadays commonly used in the ordered subset expectation maximization (OSEM) reconstruction. Correction for the attenuation is relatively simple, and both attenuation and scatter corrections have been around for a long time. However, it is challenging to accurately correct for scatter [7], and CDR correction has an tendency to introduce Gibbs artefacts [8] and to yield a different noise pattern [9]. Consequently, SPECT images suffer from poor resolution, low contrast, and high noise.

In recent years, somatostatin receptor imaging with ^68^Ga-labeled somatostatin analogues and PET/CT has improved the image quality [10] and diagnostic sensitivity [11]. Many clinical studies indicate that ^68^Ga-PET/CT is superior to ^111^In-octreotide SPECT/CT [12–17]. However, the SPECT/CT reconstruction protocols used in these studies are poorly described (Table 1). In some cases, no information is provided about the use of attenuation, scatter, or CDR corrections. Some studies even report the use of filtered back projection (FBP). One SPECT did not include CT, indicating that no corrections (even for attenuation) were made, and one states “^111^In-pentreotide imaging”, indicating that SPECT/CT was not performed at all but only a planar scintigraphy. The gathered information concludes, or in some cases indicates, that the reconstructions of the SPECT data have included sub-optimal reconstruction parameters which thereby would imply an unfair comparison. Subsequent to the majority of these studies, CDR corrections have become an alternative option in vendors’ reconstruction algorithms. The use of appropriate scatter and CDR corrections can increase SPECT image quality and hence improve the detection rate for SPECT/CT [18].

**Table 1:** Information about the reconstruction parameters and main findings reported in references (ref.)

References	Author (year)	Reconstruction information	Main findings
[11]	Lee I et al. (2015)	Iterative algorithm (8 iterations, 8 subsets)	13 patients, 35 lesions. Diagnostic sensitivity for SPECT and PET; 54% and 100%, respectively
[12]	Buchmann I et al. (2007)	Iterative algorithm, OSEM	27 patients. Visual evaluation, standard uptake values (PET), and tumor/non-tumor ratios (SPECT) were used. PET superior in lung and skeleton and similar to SPECT for liver and brain
[13]	Van Binnebeek S et al. (2016)	No information given. The SPECT did not include CT (indicating no corrections were used)	53 patients, > 1000 lesions. Sensitivity for SPECT and PET; 99.9% and 60.0%, respectively
[14]	Kowalski J et al. (2003)	Filtered back projection (FBP) with Butterworth filter, cut-off frequency = 0.6 Nyquist	4 patients. In 2 patients more findings were revealed with PET compared to SPECT
[15]	Deppen SA et al. (2016)	No information given. Only states ^111^In “imaging” (indicating that SPECT/CT was not performed but only scintigraphy)	78 scans compared. PET changed treatment in 36% of participants
[16]	Hofmann M et al. (2001)	FBP with Hanning filter, cut-off frequency = 0.5 Nyquist	8 patients, 40 lesion. PET and SPECT identified 100% and 85%, respectively
[17]	Hope T.A et al. (2019)	No information given (they refer to another study but not given there either)	150 patients. The detection rate of somatostatin receptor expressing disease was 38% and 72% for SPECT and PET, respectively

The availability of PET/CT scanners worldwide is far lower than of SPECT/CT scanners. Actually, the number of gamma cameras is over 6 times higher than PET scanners among the member countries in the Organisation for Economic Co-operation and Development (OECD) [19], even though the majority of OECD member states (34 of 38) are classified as high-income economies by the World Bank [20]. According to the World Health Organization (WHO), the proportion of countries with no PET scanner at all is 65%, compared to 35% for those with no gamma camera. Looking more specifically at the low and lower-middle World Bank income classification groups, the corresponding proportions are 94% for no PET scanners versus 48% for no gamma cameras [21]. The higher accessibility of SPECT/CT worldwide greatly increases the need for methods that improve the image quality in SPECT/CT imaging.

Vendors have implemented various analytical techniques for scatter and CDR corrections in SPECT reconstructions with the aim of enhancing image quality. Similar, or better, improvements can be achieved by using Monte Carlo (MC) simulations. MC simulations can account for the attenuation and scattering of the photons in the body as well as in the detector [22] and it can be implemented in the OSEM reconstruction of SPECT images. MC-based scatter correction has shown superior to analytical scatter correction methods such as triple-energy-window-based correction [23]. MC-based OSEM reconstruction has also shown superior in spatial resolution compared to conventional OSEM reconstruction with CDR correction [18]. Several studies have shown promising results and improved image quality with MC-based reconstruction in imaging using radionuclides such as ^99m^Tc, ^111^In, ^177^Lu, ^123^I, and ^131^I [18, 23–27]. Reconstructions using MC have previously been too time consuming for use in the clinical setting, but in 2018, Rydén et al. [25] enhanced the speed by parallelizing the simulations using graphics processing units. They presented SPECT images from phantom measurements with ^177^Lu that demonstrated clearly improved image quality within a reconstruction time of 3 min.

In this study, MC techniques will be used to simulate lesions in SPECT images, allowing us to analyze whether attenuation, scatter and CDR corrections with MC in SPECT reconstruction can improve the detection rate of liver lesions. Simulation studies often use digital phantoms with uniform activity distributions in organs [28]. However, this will not capture the influence of nonuniform activity distribution within the liver tissue. Therefore, this study incorporates patient data in the simulation of lesions, with the goal of evaluating the impact of image noise (both anatomical and statistical noise) in the detection rate of lesions. MC simulations were used to implant lesions into the raw data of patients with established (through follow up for 3 years) healthy livers. For comparison, a trained nuclear medicine specialist detected lesions in ^111^In-octreotide SPECT/CT images reconstructed with MC-based OSEM reconstruction and standard clinical attenuation-corrected (AC) OSEM.

## Methods

The Sahlgrenska Academy Reconstruction code, SARec, was used in this study to correct for attenuation, scatter, and CDR [25]. SARec uses MC simulations in the forward projection of an OSEM iterative process. It also includes a correction for the CDR function in the back projection.

Thirty-seven SPECT/CT examinations with ^111^In-octreotide, performed at the nuclear medicine department at Sahlgrenska University Hospital, Gothenburg, Sweden, between the years of 2004 and 2011, were randomly chosen. The retrospective use of the image data and waiver of consent were approved by the Regional Ethical Review Board in Gothenburg. The gamma cameras used were Millennium VG Hawkeye and Infinia Hawkeye 4 from General Electrics (GE, General Electric Medical Systems, Milwaukee, WI, USA). The crystal thickness for the Millennium VG Hawkeye was 5/8″ and for the Infinia Hawkeye was 3/8″. Medium-energy parallel-hole collimators were used. The images were acquired in 120 projections with 30 s/projection, 1 day post injection, with 110–220 MBq ^111^In-octreotide. The energy windows were two 20% windows, centered around the two photon peaks of 171 and 245 keV, respectively. The matrix size was 128 × 128 with a pixel size of 4.42 mm and a slice thickness of 4.42 mm. The matrix size of the Millennium VG Hawkeye CT was 256 × 256 with a pixel size of 2.21 mm and a slice thickness of 4.42. The matrix size of the Infinia Hawkeye 4 CT was 512 × 512 with a pixel size of 1.10 mm and a slice thickness of 5 mm.

The study population included 19 women and 18 men of mean age 52 years (range 23–84 years). The inclusion criterion was no liver lesions at the time of examination and the following 3 years. The follow up included various imaging modalities, such as CT, SPECT/CT, PET/CT, magnetic resonance imaging, and ultrasound. Raw data of simulated spheres, representing liver lesions, were implanted into the raw data of 26 of the 37 patients, one lesion per liver. The raw data of the remaining 11 patients were not modified. The spheres had a radius of 1 voxel (4.42 mm), which corresponds to a size of 4 voxels in total (volume 0.35 cm^3^) and a tumor-to-normal tissue concentration (TNC) ratio of 8. The choices of size and TNC ratio were chosen after a pre-study including the same 37 patients and spheres of two different combinations of radius and TNC ratio. In this pre-study, 13 patients received a sphere of radius 1 voxel and TNC ratio of 15, and 13 patients received a sphere of radius 1.5 voxels and TNC ratio of 5. Simulation of the sphere raw data, summation with the patient raw data and reconstructions were performed. The resulting TNC ratios in the reconstructed images were not evaluated but the visual evaluation of reconstructions with AC OSEM and MC OSEM showed that both combinations were too visible in both reconstruction methods to reflect a difference between the methods. Consequently, 37 simulated lesions with radius 1 voxel combined with TNC ratios of 5 to 10 in images of one patient reconstructed with both AC OSEM and MC OSEM were analyzed. Based on our estimates of the largest visual difference between reconstruction methods, we selected the combination of radius 1 voxel and TNC ratio of 8.

### Implementation of liver lesions

**Fig. 1:**
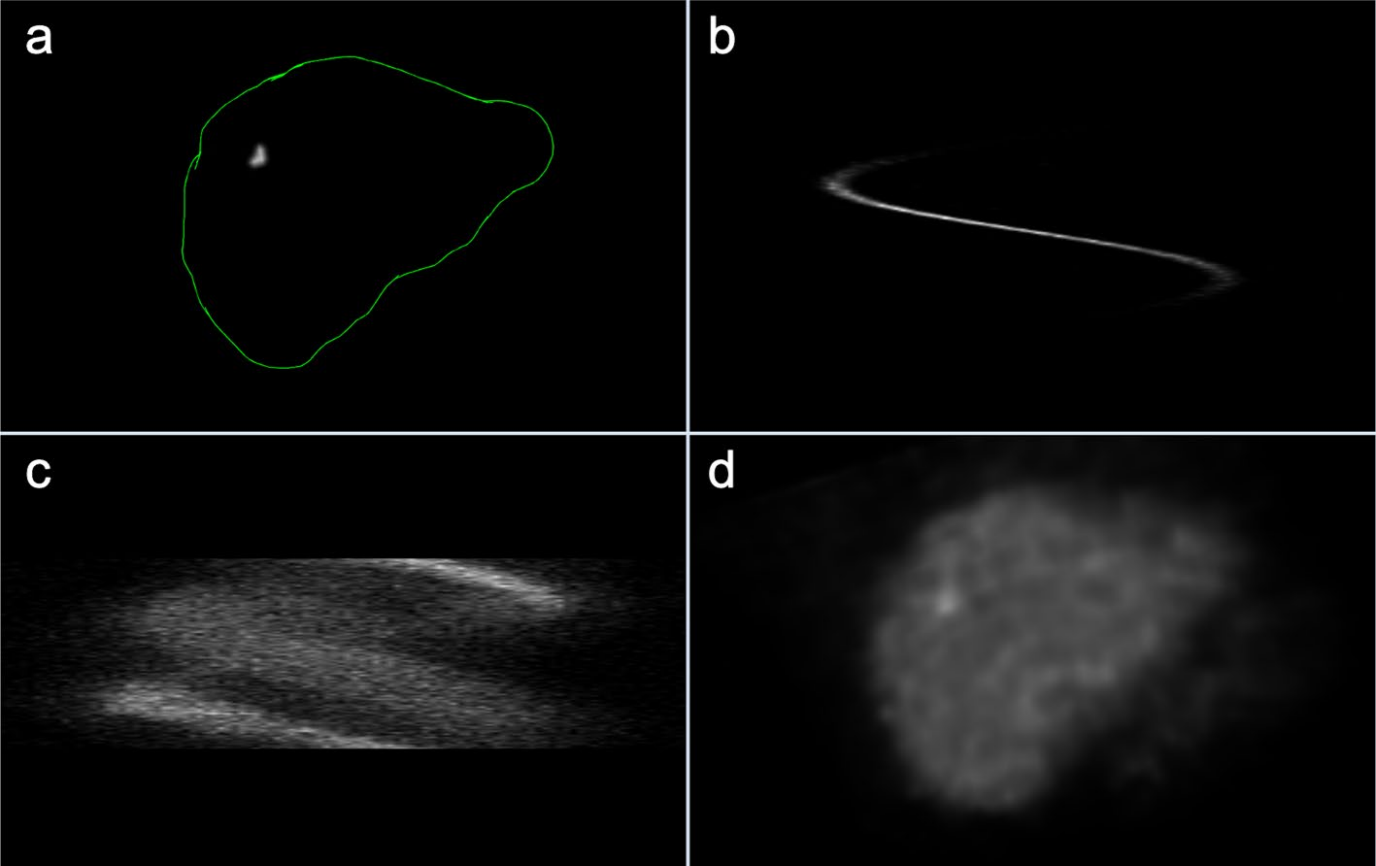
The implementation of a lesion in the liver of a healthy patient. **a** A sphere is randomly placed in the liver VOI (denoted by the green line). **b** The raw data of the lesion is generated through MC simulation of a SPECT examination. **c** The raw data of the healthy patient, which is summed with **b**. **d** The summed raw data is reconstructed with one of the reconstruction methods

A sphere (representing a lesion) was placed within the manually delineated liver volume of interest (VOI) in the reconstructed (MC OSEM) SPECT image of each patient, respectively (Fig. 1a). The position within the liver VOI was randomly determined by an in-house algorithm. To achieve a TNC ratio of 8 for the sphere compared to the surroundings, a spherical VOI, denoted “control VOI”, with a radius of three voxels (around the randomly determined position) was used to determine the local mean activity concentration in the normal liver tissue. The sphere was given an activity concentration 8 times higher than the local activity concentration. Thereafter, the sphere was used as the source in a MC simulation of a SPECT examination through the CT body of the respective patient in order to generate the sinogram of the lesion (Fig. 1b). This sinogram was then added to the sinogram of the patient (Fig. 1c), and reconstructions were made with 2 iterations and 10 subsets for standard AC OSEM (i.e., the used clinical protocol) and 5 iterations and 10 subsets for MC OSEM (Fig. 1d) [25]. This was repeated for 26 patients. The remaining 11 patients were reconstructed in the same way but from the original sinograms. Unfortunately, the vendor reconstruction application would not accept manipulated raw data, preventing us to use it for scatter and CDR corrections. Furthermore, radial distances of the detectors were not available from these acquisitions hampering the CDR correction in the vendor reconstruction even had it accepted the manipulated raw data. For the AC OSEM images, a Butterworth low-pass (LP) post filter was applied (power factor = 2 and 0.048 cycles/mm), as this is often used in the clinical setting to reduce noise. For the MC OSEM images, two sets were created, one set with no filtering and one set with a Gaussian LP post filter (standard deviation, *σ* = 3 mm). The level of smoothness (the noise level) with the Gaussian LP filter was visually chosen to be between the filtered AC (fAC) OSEM images and the unfiltered MC OSEM images. Hence, the choice of filter for MC OSEM was optimized as opposed to AC OSEM where the filter chosen was the filter used in the clinical setting at Sahlgrenska University Hospital. So, our overall aim was to compare an optimized method to the one used in the clinic. Ultimately, there were a total of 111 (3 × 37) cases for the observer to evaluate (Table 2).

**Table 2:** Three sets of (the same) 37 patients reconstructed with two different reconstruction methods and with different post filtering

	Reconstruction corrections	Post filter
fAC OSEM	Attenuation	BW LP (power factor = 2 and 0.048 cycles/mm)
fMC OSEM	Attenuation, scatter, CDR	Gaussian LP (standard deviation, *σ* = 3 mm)
MC OSEM	Attenuation, scatter, CDR	None

The sets with different combinations of reconstruction method and post filter are named filtered AC (fAC) OSEM, filtered MC (fMC) OSEM and MC OSEM

### Visual assessment

The 111 reconstructed images, including their corresponding CT images, were randomized and visually assessed by a trained nuclear medicine specialist, instructed to assess liver lesions. The observer knew there was only one potential lesion per liver. The monitor used was a 27″ Samsung SyncMaster SA850. The observer was unaware of whether the patient had a liver lesion or what type of reconstruction method or filter was used. The criteria evaluated were liver lesion yes or no with a confidence level of 1–3, where 1 was low/uncertain, 2 was moderate/probable, and 3 was high/certain. If a liver lesion was assessed, the coordinates were requested so that the accuracy could be evaluated.

### Quantitative measures

The mean activity concentration was calculated from the voxel values in the manually delineated liver VOI. The TNC ratios in the reconstructed images were computed by dividing the mean voxel value in the tumor VOI (radius of 1 voxel, volume of 4 voxels) by the mean voxel value in the normal tissue VOI. The normal tissue VOI was determined to be 34 voxels surrounding the tumor voxels in all three planes (transverse, sagittal, and coronal), not counting the voxels adjoined to the tumor VOI (due to the partial volume effect). Signal-to-noise ratios (SNRs) were calculated, with the signal represented by the mean voxel value in the tumor VOI and the noise represented by the standard deviation of the mean voxel value in 20 randomly placed (in the liver) VOIs of the same size as the tumor VOI (4 voxels). The percentage of detected lesions and accuracy were manually calculated. Accuracy was determined as the percentage of correctly classified cases (i.e., correctly classified lesions, including correct coordinates, or correctly classified with no lesions). Receiver operating characteristic (ROC) analyses were performed, with a rating scale of 1–6 transformed from the observer’s assessment of lesion yes (1) or no (0) and confidence level (1–3).

### Statistical analyses

ROC curves, analyses, and statistical tests were compiled with the software IBM SPSS Statistics for Macintosh, Version 27.0 (IBM Corporation, Armonk, New York, USA). Statistical tests used were independent samples *T* test and one-way ANOVA repeated measures with Bonferroni adjustment for multiple comparisons. A *p* value less than 0.05 was considered to indicate statistical significance.

## Results

### Visual assessment and quantitative measures

**Fig. 2:**
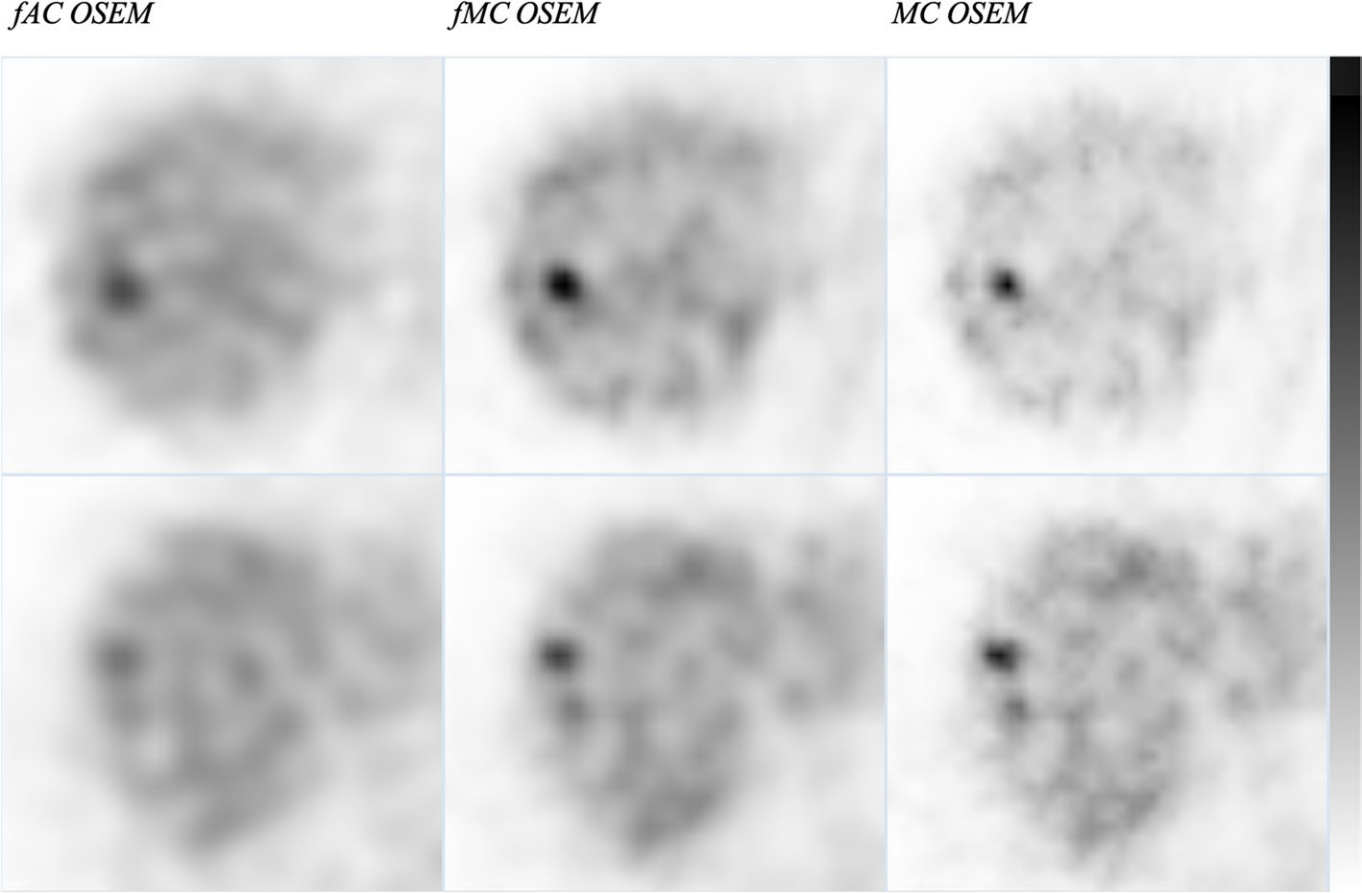
Examples of a lesion that was detected in all reconstruction methods (upper row) and another lesion that was detected in fMC OSEM and MC OSEM but not in fAC OSEM (lower row)

The detection rate for fAC OSEM was 30.8% and increased to 50.0% when scatter and CDR corrections with MC was implemented in the SPECT reconstruction (MC OSEM) (Table 3). Reducing the noise level in MC OSEM by adding post filtering (fMC OSEM) reduced the detection rate to 42.3%. Figure 2 shows an example of a lesion that was detected in all reconstruction methods and another lesion that was detected in fMC OSEM and MC OSEM but not in fAC OSEM. The accuracy of 45.9% for fAC OSEM and fMC OSEM and 54.1% for MC OSEM (Table 3) differs from the detection of lesions since it also considers the accuracy of assessing the healthy patients with no lesions; hence, all 37 patients are included. There were more false positive findings on case level for fMC OSEM and MC OSEM (*n* = 5 and *n* = 4) than for AC OSEM (*n* = 2), which influences the accuracy. On lesion level (including false positive findings in livers where the observer found the wrong lesion) the number of false positive findings were 4 for fAC OSEM, 10 for fMC OSEM and 5 for MC OSEM.

**Table 3:** The detection of lesions and accuracy for all lesions (AL) and detectable lesions (DL) for the three reconstruction methods

Kolumn1	Scenario	fAC OSEM	fMC OSEM	MC OSEM
Detection of lesions (%)	AL	30.8	42.3	50.0
	DL	42.1	57.9	68.4
Accuracy (%)	AL	45.9	45.9	54.1
	DL	59.5	62.2	73.0

The modest detection rate was partially attributed to the pronounced partial volume effect; the TNC ratios, after reconstruction, for the non-detectable lesions were reduced from 8 to mean values of 1.12, 1.22, and 1.28 for fAC OSEM, fMC OSEM, and MC OSEM, respectively (Table 4). The corresponding mean TNC ratios for the detectable lesions were 1.19, 1.45, and 1.73. There was a significant statistical difference in TNC ratios between non-detectable and detectable lesions for all reconstruction methods (Table 4). There were also statistically significant differences (*p* < 0.001) between the TNC ratios (for all lesions) between the three reconstruction methods, fAC OSEM–fMC OSEM, fMC OSEM–MC OSEM, and fAC OSEM–MC OSEM. The SNR in the liver was significantly higher, statistically, in detectable lesions compared to non-detectable lesions: 11.3 versus 8.2 for fAC OSEM, 12.8 versus 7.9 for fMC OSEM, and 12.3 versus 7.6 for MC OSEM (Table 4). For SNR, there was no statistical difference between any of the reconstruction methods. There was not any statistically significant difference in the mean activity concentration in livers with detectable and non-detectable lesions for any of the reconstruction methods. Nor was there any statistically significant difference in the mean activity concentration in livers that had false positive findings and those that had not. The mean TNC ratios (ranges) for false positive lesions were 1.16 (1.09–1.27) for fAC OSEM, 1.43 (1.21–1.72) for fMC OSEM and 1.91 (1.59–2.25) for MC OSEM.

**Table 4:** The mean TNC ratios (ranges) and mean SNRs (ranges) of the non-detectable and detectable liver lesions for each reconstruction method with corresponding *p* values and 95% confidence interval (CI)

	Non-detectable	Detectable	*p* value (95% CI)
Mean TNC ratio (range)			
fAC OSEM	1.12 (0.96–1.25)	1.19 (1.14–1.23)	.015 (0.02–0.13)
fMC OSEM	1.22 (0.83–1.57)	1.45 (1.25–1.85)	.005 (0.08–0.39)
MC OSEM	1.28 (0.83–1.96)	1.73 (1.44–2.55)	.002 (0.19–0.70)
Mean SNR (range)			
fAC OSEM	8.2 (3.2–14.0)	11.3 (7.7–18.3)	.018 (0.59–5.71)
fMC OSEM	7.9 (3.3–14.8)	12.8 (7.9–18.4)	< .001 (2.30–7.45)
MC OSEM	7.6 (2.9–18.9)	12.3 (7.8–18.1)	.004 (1.62–7.77)

**Fig. 3:**
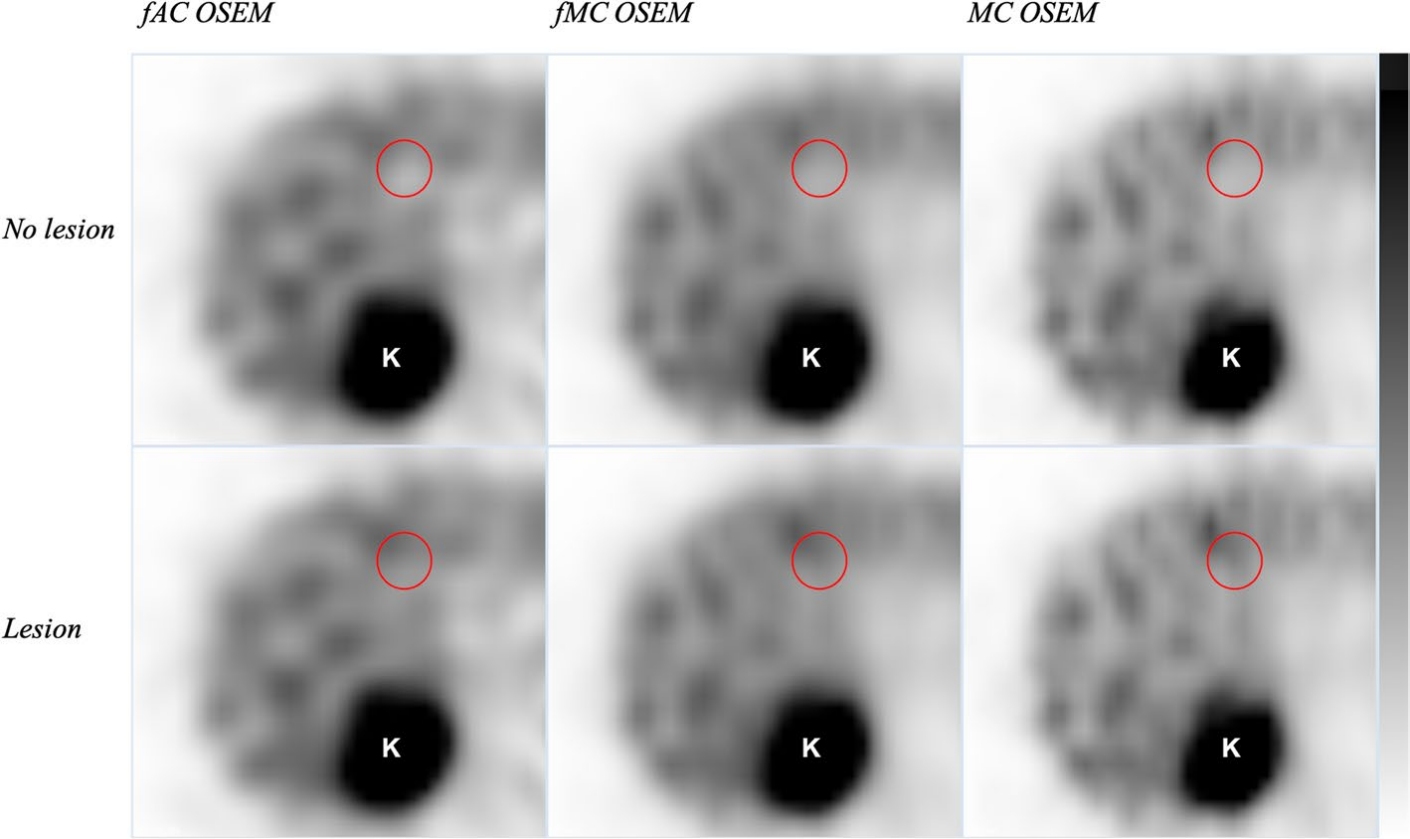
Visual demonstration of the disappearance of simulated lesions when placed in a region of low activity concentration. The upper and lower rows show reconstructions without and with the simulated lesion, position denoted by the red circle, respectively. K denotes the right kidney. From left to right: fAC OSEM, fMC OSEM, and MC OSEM

Retrospective visual inspection demonstrated that lesions randomly simulated into liver regions with low activity concentration were not visually detectable after reconstruction (Fig. 3). A total of 7 patients were judged by two observers to have simulated lesions that could not be considered detectable. The mean TNC ratios and mean SNRs of these lesions were 1.05 (range 0.96–1.12) and 6.1 (3.2–9.4) for fAC OSEM, 1.04 (0.83–1.20) and 5.7 (3.3–8.9) for fMC OSEM, and 1.04 (0.83–1.25) and 5.0 (2.9–7.4) for MC OSEM. Results are therefore presented for two situations: (1) assuming that all simulated lesions are detectable, scenario “all lesions” (AL); and (2) assuming that only the lesions possible to detect are considered lesions, scenario “detectable lesions” (DL). The detection of lesions and accuracy have already been presented for scenario AL (Table 3). With 7 patients changed to be negative for lesion involvement, scenario DL, the detection of lesions and accuracy increased (Table 3). Of 19 simulated lesions, the observer detected 42.1% with fAC OSEM, 57.8% with fMC OSEM, and 68.4% with MC OSEM. The accuracies were 59.5%, 62.2%, and 73.0% for fAC OSEM, fMC OSEM, and MC OSEM, respectively.

### ROC analysis

**Fig. 4:**
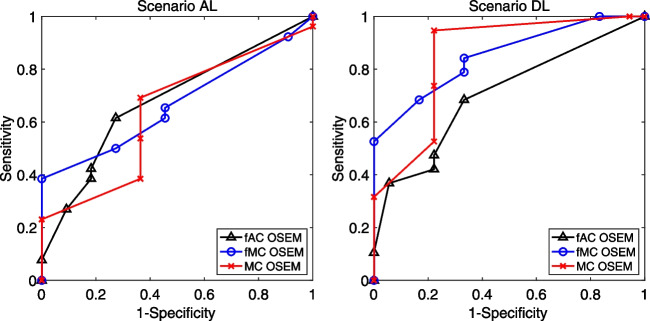
ROC curves for all reconstruction methods and for scenario (left) AL and (right) DL. Black triangles—fAC OSEM, blue circles—fMC OSEM, and red crosses—MC OSEM

ROC curves for scenarios AL and DL are shown in Fig. 4. ROC areas under the curve (AUCs) for each reconstruction method and for scenarios AL and DL are presented in Table 5. Note that the ROC analysis does not consider the coordinates of the lesions, which may result in discrepancies between the ROC analysis and the detection of lesions/accuracy of assessments. Misplacement, meaning a liver lesion with incorrect coordinates (a false positive finding in a liver where the true lesion was missed), of lesions was present for all reconstruction methods in scenario AL and for fMC OSEM and MC OSEM in scenario DL (Table 6). So, between the reconstruction methods, more misplaced lesions led to better outcomes in the ROC analysis (since a misplaced lesion is considered an accurate assessment). A deeper analysis showed that, for fAC OSEM, both patients for whom the observer misplaced the lesion had lesions that disappeared; therefore, they became false positives in scenario DL. For fMC OSEM, this happened in one of five patients, and for MC OSEM, the patient with the misplaced lesion was not in the group where the lesion disappeared. However, it is important to note that no statistically significant differences between the AUC for any of the reconstruction methods, for any scenario, were found.

**Table 5:** ROC area (95% CI) under the curve for the three reconstruction methods and for scenario AL and DL, respectively

Fitted ROC area	fAC OSEM	fMC OSEM	MC OSEM
Scenario AL	0.680 (0.496–0.864)	0.668 (0.495–0.841)	0.638 (0.444–0.832)
Scenario DL	0.705 (0.536–0.873)	0.851 (0.729–0.973)	0.852 (0.723–0.982)

**Table 6:** Number of patients where the lesion was misplaced (assessed as positive for lesion but inaccurate coordinates) for scenario AL and DL, respectively

	AL	DL	Comment
fAC OSEM	2	0	Both patients became false positive in scenario DL
fMC OSEM	5	4	One of these patients became false positive in scenario DL
MC OSEM	1	1	This patient was not changed between scenarios AL and DL

### Confidence level

**Fig. 5:**
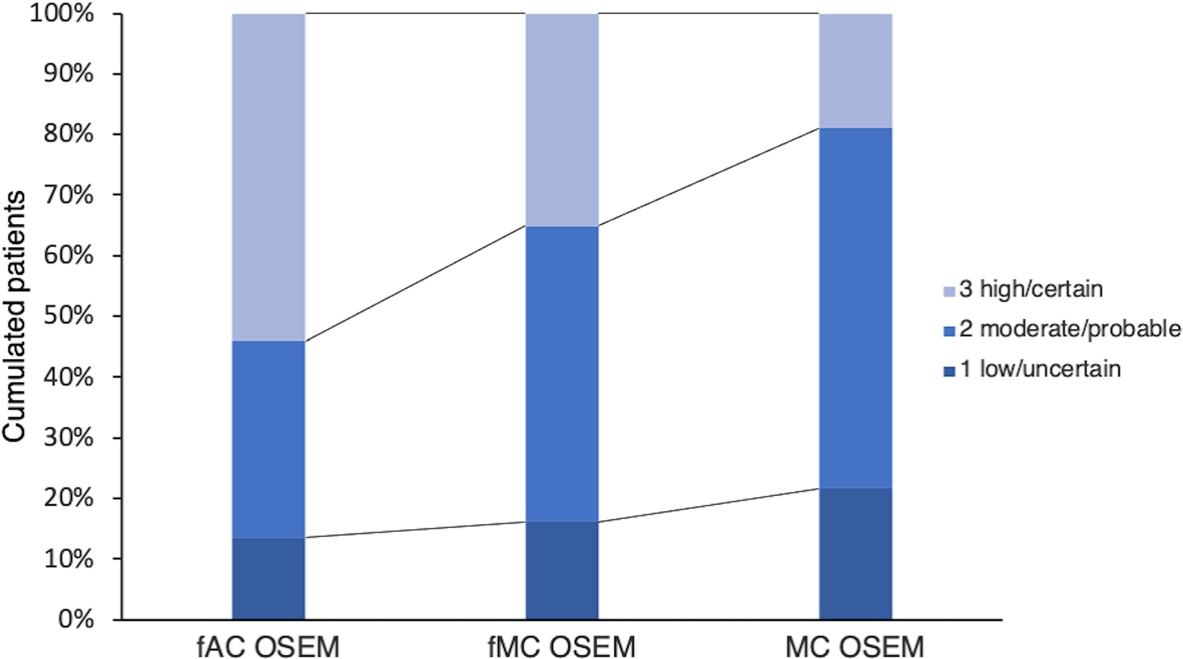
The confidence level of the observer in the three sets of images

**Fig. 6:**
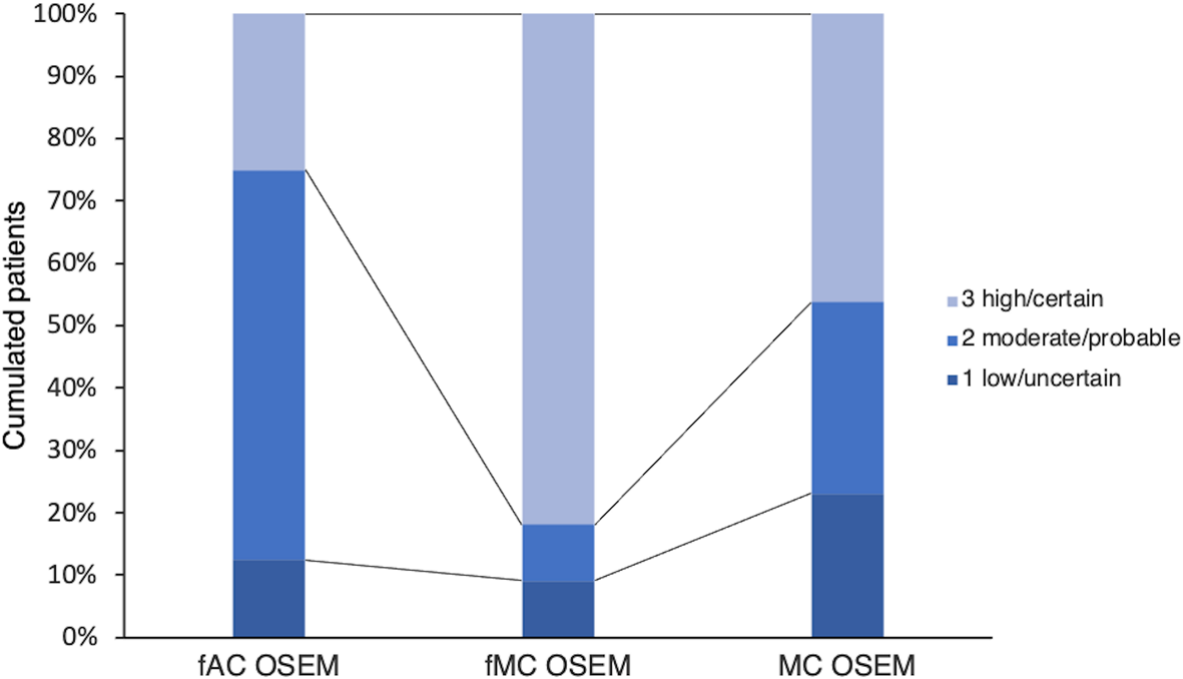
The confidence level of the observer, regarding detectable lesions, in the three sets of images

The highest confidence level of the observer (high/certain) was used most frequently in the fAC OSEM images and more frequently in the filtered MC OSEM images compared to the unfiltered images (Fig. 5). However, for detectable lesions only, there was a higher confidence level for fMC OSEM and to some extent also for MC OSEM compared to fAC OSEM (Fig. 6).

## Discussion

This study, in which lesions were randomly simulated into the liver tissue using clinical raw data from SPECT acquisitions, demonstrated that the detectability of the simulated lesions is highly dependent on the local activity estimate and the noise level. The lesions that were detectable for the observer had statistically significant higher TNC ratios and SNR. Using MC-based OSEM reconstruction, the spatial resolution was improved [18] and consequently the TNC ratios were statistically significantly improved compared to conventional reconstruction (fAC OSEM). Although, the noise level remained high, which influenced the SNR values.

For all reconstruction methods, the variation in liver activity concentration and high noise level resulted in disappearance of lesions when the randomly selected location was in an area of low activity concentration, about the size of the control VOI (Fig. 3). These lesions received a lower TNC ratio due to the low activity concentration of the control VOI, and there was a higher activity concentration outside of the control VOI. The volume of the lesions after reconstruction was, due to poor spatial resolution, approximately the same size as the control VOI and the partial volume effect consequently spread the activity in the lesion over the area with low activity concentration. These lesions had low TNC ratios and the visual retrospective assessment, with knowledge of the correct coordinates, resulted in the decision to consider these patients negative for lesions.

Since several lesions were considered not detectable after the retrospective analysis, we also performed an analysis of only the lesions possible to detect. Our viewpoint was that this scenario more fairly demonstrates the differences between the three reconstruction methods. The hidden lesions were naturally not detected by the observer in any of the reconstruction methods in scenario AL. Therefore, the difference between the reconstruction methods in scenarios AL and DL regarding the detection of lesions was only a reflection of the lower number of patients that are lesion positive (26 for each set in AL vs. 19 in DL). There were a larger number of misplaced lesions in scenario AL than in scenario DL (Table 6). Therefore, the ROC analysis differed more from the results of the detection of lesions and the accuracy of the assessments in scenario AL than in scenario DL. Furthermore, the misplaced lesions that had an erroneous positive impact in the ROC analysis did not influence the detection of lesions measure but did affect the accuracy in scenario AL. When 7 patients were changed to negative for lesion, some of these misplacements shifted (rightfully) to false positives, which explained the differences in accuracy between scenarios AL and DL. Another feature of ROC analysis is that it considers the confidence level of the observer. Figure 5 shows that the confidence level was higher in filtered images compared to unfiltered images and highest in fAC OSEM. This was possibly due to the observer’s previous experience with image appearance during evaluations.

According to a previous study [18], the spatial resolution is improved in images reconstructed with MC OSEM, which would consequently imply a higher degree of detail. The perception of homogeneity of the uptake of ^111^In-octreotide in the liver, which is usually what the observer expects in a SPECT/CT examination, can be challenged as the spatial resolution and image quality overall improve. The blurring effect of commonly used filters also contributes to the homogenous appearance. This resembles situations in which new gamma camera designs present higher sensitivity and/or resolution. Pathologic uptake will be more intensive, but this also applies for benign findings; for all reconstruction methods the TNC ratios of the false positive findings were similar to the TNC ratios of the detected lesions. This might explain the higher degree of false positive findings by the observer in fMC OSEM and MC OSEM in this study. Further, the images reconstructed with MC OSEM (specifically unfiltered) were very different visually from those most familiar to observers, which is a probable explanation for the lower confidence level reported by the observer. In this study, the observer was more confident with smoother images (Fig. 5). However, the confidence level of the observer regarding the detected lesions was highest with fMC OSEM (Fig. 6), a probable cause of higher TNC ratios combined with low noise levels. Still, the detection of lesions and the observer accuracy were improved with unfiltered MC OSEM compared to the filtered images (Table 3).

As stated before, the ROC analysis did not consider the coordinates of the lesion, so a misplaced lesion in a patient who has a lesion was considered an accurate assessment. It can be argued that this is a correct approach when evaluating a diagnostic test for a disease, since the underlying reason for a positive test is irrelevant and the goal is to find patients who require further evaluation. In this study, however, we aimed to distinguish differences in the visual appearance of small lesions depending on the different image processing techniques. From our perspective, a misplaced lesion is due to two successive errors: the first misses the real lesion and the second finds a lesion that is not there. To consider this to be an accurate assessment is therefore unsatisfactory. Consequently, the terms detection of lesions and accuracy were chosen instead of sensitivity and specificity. As there were misplaced lesions for all reconstruction methods, and in the absence of other comparison measures, the ROC analysis was still considered valuable. This was especially true for scenario DL, where there were not as many misplaced lesions as for scenario AL. There is an alternative to ROC, called Free response operating characteristic (FROC), that does consider the position of the lesion. [29, 30] However, our study was not designed as a FROC study which made it difficult to analyze the data. The assessment in FROC (the detection as well as the confidence level) is made on lesion level instead of case level. Also, using Jackknife FROC analysis (JAFROC and JAFROC1) we could still not handle the problem with misplaced lesions (where the observer found a lesion but with the wrong coordinates) as JAFROC only considers false positives in normal cases and JAFROC1 does but recommends to not include normal cases at all.

The CT scanners used for SPECT/CT imaging at Sahlgrenska University Hospital during the years between 2004 and 2011 were, from an image quality standpoint, far inferior to the CT scanners used today. The image quality of the CT images in this study was poor, and some examinations also suffer from severe metal artifacts; these will influence the performance of the MC simulations in the reconstruction algorithm. In conventional OSEM reconstruction, the CT images are used only for attenuation correction. Hence in this study, the image quality of the CT was of less importance for fAC OSEM than for fMC OSEM and MC OSEM. Furthermore, the radial positions of the detectors at each projection angle were not registered by the gamma cameras. Therefore, the distances, in order to correct for the CDR function, had to be manually estimated (based on the CT images), which might have influenced the accuracy of the MC simulations. Hence, higher quality CT images and registered radial distances by the gamma camera, both standard in SPECT/CT scanners today, might result in more accurate MC simulations, and consequently the image quality might be further improved.

The post filtered AC OSEM reconstruction used in this study includes all the parameters that were used when the examination was performed at Sahlgrenska University Hospital, it has not been optimized. Today, there are commercially available reconstruction methods that apply CDR correction. To include this correction in AC OSEM would have been an interesting comparison. At Sahlgrenska University Hospital, we have access to GE’s reconstruction application with CDR correction, called Evolution, but it did not accept our manipulated raw data, nor was there any information registered by the gamma cameras of the radial distances, which is required to perform the correction. However, we previously compared the spatial resolution in ^111^In octreotide imaging and showed a statistically significant improvement in images reconstructed with MC OSEM versus Evolution (8.2 mm vs. 9.3 mm, *p* < 0.001; 95% CI 0.6–1.5) [18]. The use of CDR correction in conventional reconstruction, fAC OSEM, might probably have improved these images but as the spatial resolution improvement in MC OSEM compared to Evolution was statistically significant, MC OSEM would still be expected to be superior. However, this had to be evaluated in a comparative study. Furthermore, the number of observers should ideally be more than one.

It has previously been shown that MC OSEM significantly improves the image quality in ^177^Lu-octreotate imaging [25] and the spatial resolution in ^111^In-octreotide imaging [18]. As MC simulations can be performed for all energies, the image quality in MC-based reconstruction is improved also for imaging with other radionuclides like the work horse ^99m^Tc [23] and also ^90^Y [31]. MC-based reconstructions are also favorable for radionuclides with higher photon energy components, that cause problems with scatter, which has been shown in phantom studies with ^123^I and ^131^I [26, 27]. Hence, MC-based reconstruction is very promising. However, the noise level needs to be handled appropriately, and we aim to further investigate deep learning–generated synthetic intermediate projections (SIPs) in SPECT images, which have been demonstrated to more effectively reduce the noise level compared to post-filtering methods such as Gaussian filtering [32, 33]. This might improve SNR in images reconstructed with MC-based OSEM reconstruction.

## Conclusion

This study demonstrates that the detection rate of liver lesions is highly dependent on the image noise and resolution. Introduction of scatter and CDR correction with MC simulations will improve the detectability, but further improvement of noise reduction techniques is warranted.

## Data Availability

The data used in this study are available from the corresponding author upon reasonable request.

## Abbreviations

AC: Attenuation corrected
AL: All lesions
AUC: Area under the curve
CDR: Collimator detector response
CI: Confidence interval
CT: Computed tomography
DL: Detectable lesions
fAC: Filtered attenuation corrected
FBP: Filtered back projection
FROC: Free response operating characteristic
fMC: Filtered Monte Carlo
JAFROC: Jackknife free response operating characteristic
LP: Low pass
MC: Monte Carlo
NET: Neuroendocrine tumor
OECD: Organisation for Economic Co-operation and Development
OSEM: Ordered subset expectation maximization
PET: Positron emission tomography
ROC: Receiver operating characteristic
SIP: Synthetic intermediate projections
SNR: Signal-to-noise ratio
SPECT: Single-photon emission computed tomography
TNC: Tumor-to-normal tissue concentration
VOI: Volume of interest
WHO: World Health Organization
